# Band Gap Engineering of Hexagonal SnSe_2_ Nanostructured Thin Films for Infra-Red Photodetection

**DOI:** 10.1038/s41598-017-15519-x

**Published:** 2017-11-09

**Authors:** Emma P. Mukhokosi, Saluru B. Krupanidhi, Karuna K. Nanda

**Affiliations:** Materials Research Center, Indian Institute of Science, Bangalore, 560012 India

## Abstract

We, for the first time, provide the experimental demonstration on the band gap engineering of layered hexagonal SnSe_2_ nanostructured thin films by varying the thickness. For 50 nm thick film, the band gap is ~2.04 eV similar to that of monolayer, whereas the band gap is approximately ~1.2 eV similar to that of bulk for the 1200 nm thick film. The variation of the band gap is consistent with the the theoretically predicted layer-dependent band gap of SnSe_2_. Interestingly, the 400–1200 nm thick films were sensitiveto 1064 nm laser iradiation and the sensitivity increases almost exponentiallly with thickness, while films with 50–140 nm thick are insensitive which is due to the fact that the band gap of thinner films is greater than the energy corresponding to 1064 nm. Over all, our results establish the possibility of engineering the band gap of SnSe_2_ layered structures by simply controlling the thickness of the film to absorb a wide range of electromagnetic radiation from infra-red to visible range.

## Introduction

Single and few layered metal dichalogenide materials such as WS_2_, MoS_2_, MoSe_2_, MoTe_2_, TaS_2_ have been widely explored to develop various electronic devices such as transistors^1^, photo-detectors^2^, energy storage^3^, and humidity sensors^4–6^, etc because of their unique layer dependent structural and electronic properties such as tunable band gaps^4–9^. Among the layered materials, SnSe_2_ is an earth abundant n-type binary semiconductor whose band gap can be tuned for a wide range of electromagnetic spectrum from 1–2 eV making SnSe_2_ attractive material for various elctronic device applications^7^. The semiconducting nature of SnSe_2_ was discoveved in 1955 during an investigation into the fundamental factors that are responsible for intrinsic semiconductivity in certain intermetallic compounds and compounds formed by the metalloids Se and Te^8^. The carrier concentration varies from 10^17^–10^19^ cm^−3^, electron mobilities µ_e_ between 0.6–85 cm^2^/V s^9–15^, a direct and indirect band gap between 0.9–2.04 eV^13,16–18^, high absorption coefficient of >10^4^ cm^−1^. It crystallizes in the CdI_2_ hexagonal lattice^19^ and is a prototype of transitional metal dichalogenides, composed of two-dimensional Se-Sn-Se sheets stacked on the top of one another and characterized by strong covalent bonding between Se-Sn-Se atoms and week interlayer Van der Waal’s bonding.

SnSe_2_ thin films have been prepared by spin coating^20,21^, spray pyrolysis^14,22,23^, chemical vapour deposition(CVD)^15,18^, molecular beam epitaxy^10^, thermal evaporation of Sn and Se elements^24^, and sputtering^25^. Here, we deposited Sn films of various thicknesses on soda lime glass (SLG) substrate and annealed the films in selenium atmosphere for 1 h at 450 °C to obtain hexagonal SnSe_2_. Interestingly, the band gap varied from 2.04 eV for 50 nm thick film to 1.20 eV for 1200 nm thick one. Furthermore, 400–1200 nm thick films were shown to be sensitive to 1064 nm laser iradiation, while films with 50–140 nm thick is insensitive which is due to the fact that the band gap of thinner films is greater than the energy corresponding to 1064 nm. Over all, our results establish the possibilty of engineering the band gap of SnSe_2_ layered structure by simply controlling the thickness of the film to absorb a wide range of electromagnetic radiation from infra-red to visible range.

## Results and Discussion

**Figure 1:**
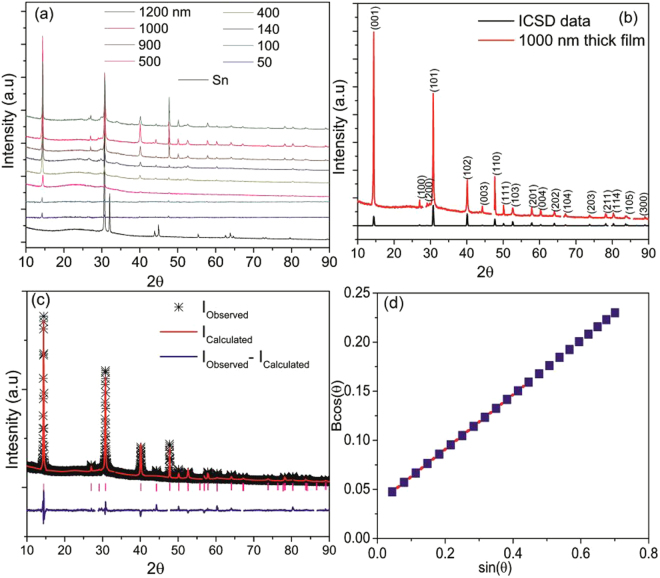
(**a**) XRD patterns of SnSe_2_ films, (**b**) comparison of 1000 nm thick SnSe_2_ film with ICSD data, (**c**) Rietveld refinement, and (**d**) Williamson-Hall plot of 1200 nm thick SnSe_2_ film.

The thickness of the deposited Sn films on SLG varied from 30–600 nm and after selenisation, it increased to 50–1200 as shown in Figure S1 (Supporting Information) and were measured using Dektak profilometer (deviation is ± 10 nm) and Atomic force microscopy (AFM) for 50 nm thick film only. The overall thickness of SnSe_2_ films is almost double the thickness of the Sn films. X-ray diffraction (XRD) patterns of SnSe_2_ thin films are shown in Fig. 1a and matches well with the ICSD data as evident from Fig. 1b. It is observed that hexagonal pure phase of SnSe_2_ with space group P-3 m1 was formed with no evidence of any secondary phases. Rietveld refinement using GSAS^26^ software program was used to fit the crystal structure for the 1000 thick film as shown in Fig. 1c. The criteria for fitting is based on least squares refinement theory which gives indicators of the quality of refinement (**SI**). The details of the least squares refinement and associated parameters obtained in this study are shown in SI (Tables S1 and S2). The fitting parameters yield *a* = *b* = 0.3818 nm and *c* = 0.6152 nm with a strain of 0.12% from the Williamson-Hall plot (Fig. 1d).

Figure S2 shows the room temperature Raman spectra of SnSe_2_ films. From Figure S2b, two Raman active modes at 115 and 183.5 cm^−1^ are observed which belong to SnSe_2_ hexagonal phase^6,10,19,25,27,28^. The peak at 115 cm^−1^ is for E_g_ mode and is due to in-plane strecthing, while the peak located at 183.5 cm^−1^ is for A_1g_ mode due to out of plane stretching of selenium atoms^29^. The intensity of A_1g_ mode increases with the thickness (Figure S2c) as is the case of layer dependency^7^.

**Figure 2:**
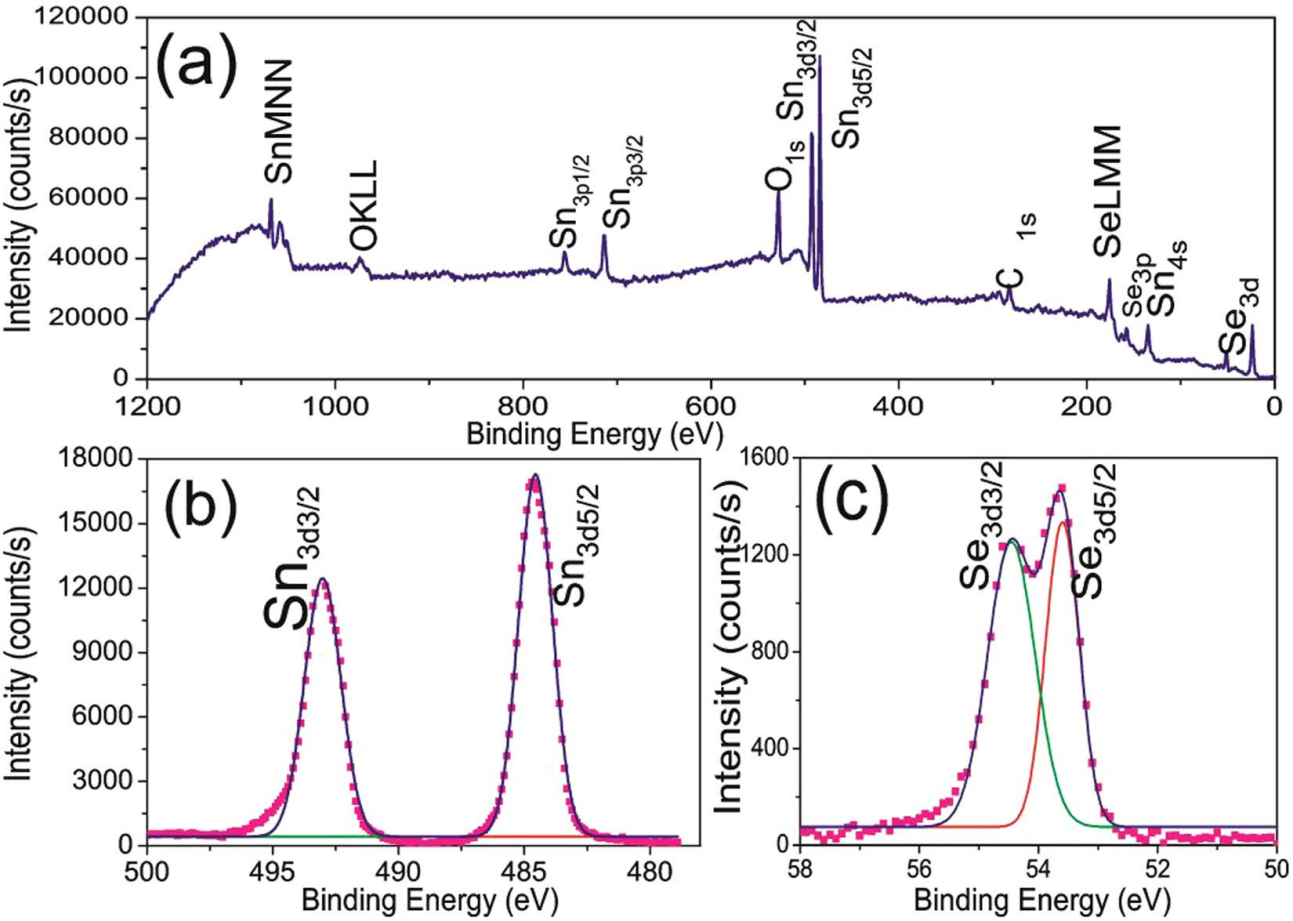
(**a–c**) Typical XPS survey, Se and Sn spectra. No presence of other oxidation states is realized.

In order to investigate the chemical electronic states of the prepared SnSe_2_ thin films, x-ray photoelectron spectroscopy (XPS) study was performed. In the survey spectrum of SnSe_2_, the peaks corresponding to Sn_3d_, C_1s_ and Se_3d_ were identified as shown in Fig. 2a. In addition, O peak observed may be from the SLG substrate. The spectra of Sn_3d_ and Se_3d_ were measured to determine the oxidation states of the constituent elements. The spectra of the constituent elements is shown in Fig. 2b and c. Sn_3d_ state splits into two states of Sn_3d3/2_ and Sn_3d5/2_ with binding energy of 493.01 and 484.55 eV respectively with a peak to peak separation of 8.46 eV, which is an indication of Sn^4+^ ^30–32^. The peaks at 54.46 and 53.59 eV are related to Se_3d3/2_ and Se_3d5/2_ states, respectively^30–34^. Figure S3 shows the energy dispersive x-ray (EDX) spectrum of 1200 nm thick SnSe_2_ films. The presence of Cu and C in the spectrum emerges from carbon coated copper grids. We can conclude that the SnSe_2_ thin films on SLG, confirms the oxidation states of constituent elements of Sn and Se with no presence of other oxidation states.

**Figure 3:**
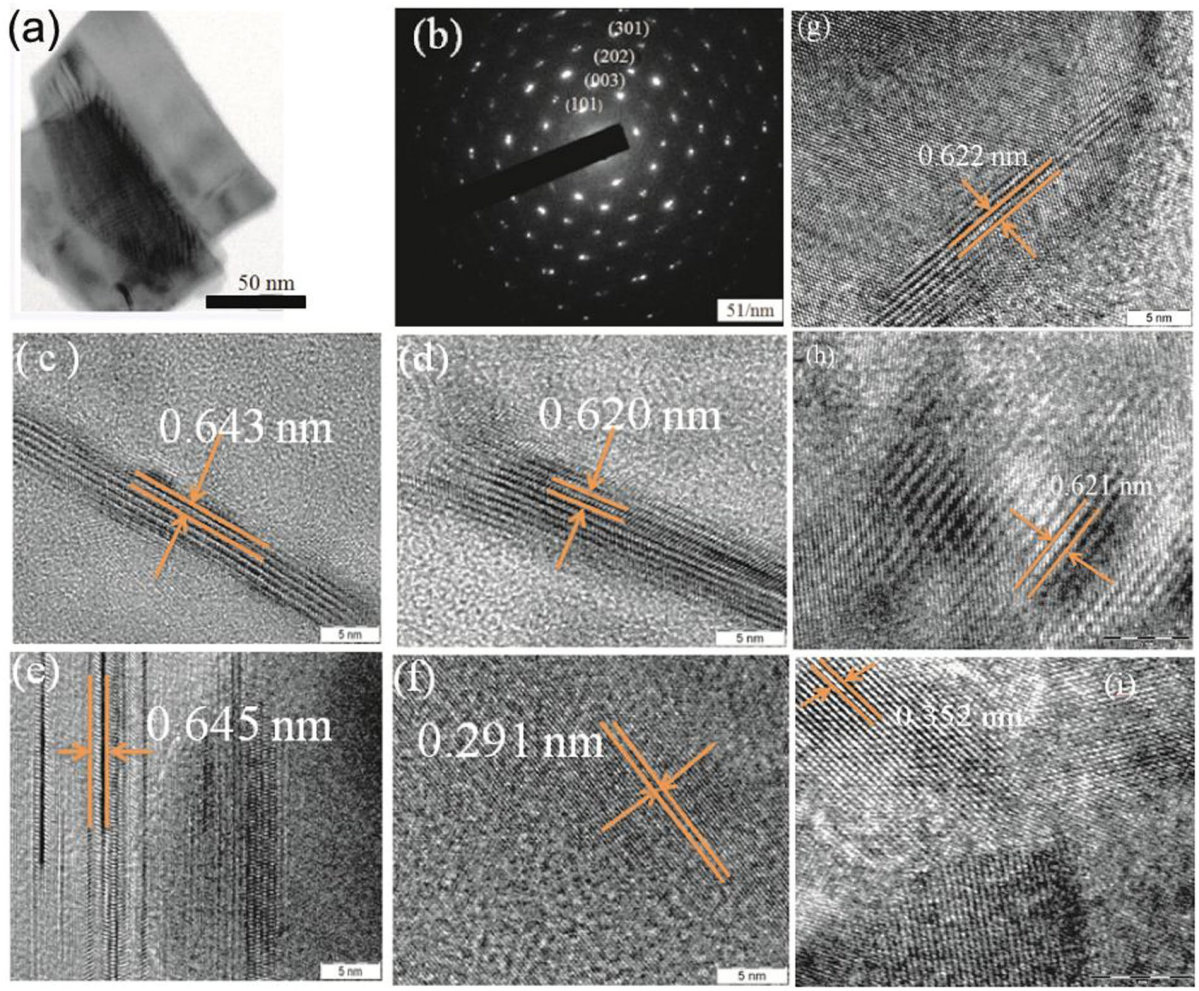
(**a**) TEM image and (**b**) SAED pattern and (**c**) HRTEM image of a 1000 nm thick SnSe_2_ film. (**d,e**) HRTEM images of a 1200 nm thick film. HRTEM images of (**g**) 900 nm, (**h**) 400 nm and (**i**) 140 nm thick SnSe_2_ films.

Transmission electron microscope (TEM), high resolution TEM (HRTEM) images and selected area electron diffraction (SAED) pattern shown in Fig. 3(a–f) confirm SnSe_2_ hexagonal crystal structure. The strong reflections in SAED pattern shown in Fig. 3b and HRTEM fringes presented in Fig. 3c and d strongly confirm further the high cystallinity and layered hexagonal crystal structure of SnSe_2_ with inter layer distances of about 0.643 nm^15^. The obtained fringes of ~0.6430 and 0.291 nm in HRTEM images shown in Fig. 3(c–e) and f correspond to (001) and (100) crystal plane of the hexagonal crystal system^35,36^. In the SAED pattern (Fig. 3b), the crystal planes of (101), (003), (202) and (301) correspond to d-spacings of 0.2894, 0.2100, 0.1475 and 0. 1034 nm, respectively of the hexagonal crystal system with space group P-3m1^18,37,38^. These results are consistent with XRD planes for the hexagonal crystal system with space group P-3m1 shown in Fig. 1b. HRTEM images shown in Fig. 3(g–i) for 900, 400 and 140 nm thick films also show similar features as other thicknesses. The diffraction spots in SAED of Fig. 3b appear elongated and may suggest the formation of small crystalline domains inside the SnSe_2_ layers which are disoriented with respect to each other^39,40^.

**Figure 4:**
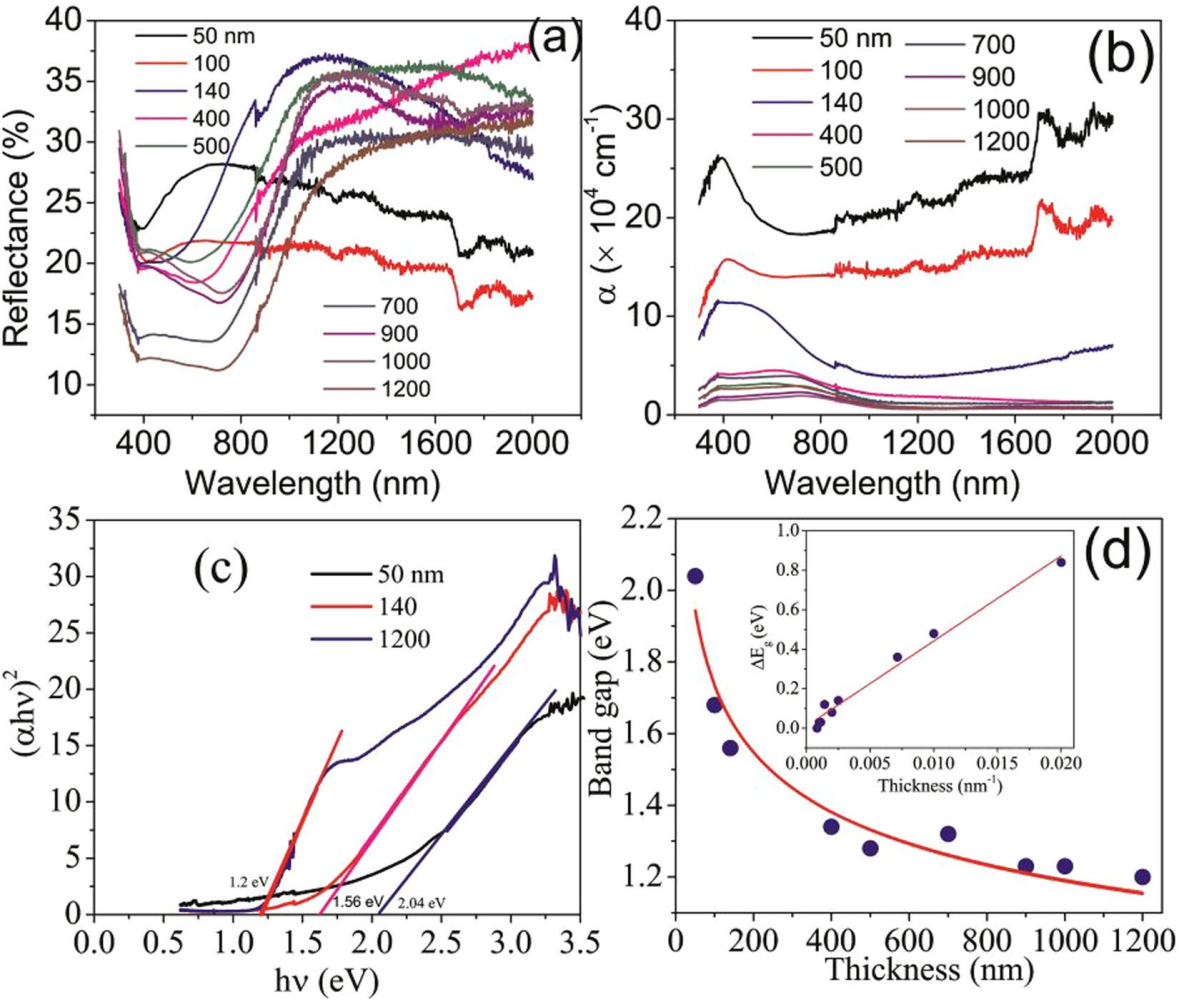
(**a**) DRS of SnSe_2_ thin films on SLG substrate of various thicknesses, (**b**) absorbance versus wavelength of SnSe_2_ thin films, (**c**) Tauc plot for 50, 140 and 1200 nm thin films, and (**d**) variation of band gap with thickness. Inset shows the change in band gap as a function of the inverse of the thickness. The solid lines are guide to the eye.

The optical properties of SnSe_2_ thin films on SLG substrate were evaluated by taking diffuse reflectance spectra (DRS) between 300–2000 nm of wavelength as shown in Fig. 4a. Using the Kubelka-Munk (KM) function^41^, the DRS was then converted to an equivalent absorption spectra as shown in Fig. 4b. The KM function at any wavelength is given by $F({R}_{\infty })=\frac{{(1-{R}_{\infty })}^{2}}{2{R}_{\infty }}=\frac{\alpha }{S}$, where ${R}_{\infty }$ is the reflectance of the film relative to the reference material (i.e. $\frac{{R}_{sample}}{{R}_{reference}}$), α is the absorption coefficient and S is the scattering coefficient. The scattering coefficient is weakly dependent on energyand therefore, $F({R}_{\infty })$ is assumed to be proportional to the absorption^42^. The optical band gap of SnSe_2_ thin films were then estimated from Tauc plot^43^ as shown in Fig. 4c and Figure S4 *i.e*. a plot of (αhυ)^2^ against hυ for direct band gap material and the variation in band gap with thickness is shown in Fig. 4d. From Fig. 4d, it can be noted that the band gap depends on the thickness of the film which in turn depends on the number of layers^7^. It is interesting to note that the variation of energy corresponding to maximum absorption is similar to that of band gap (Figure S5) and the band gap varies linearly with the inverse of the thickness (inset of Fig. 4d). For 50 nm thick film, the band approximates 2.04 eV similar to that of monolayer thick film, whereas the band gap is approximately 1.2 eV for the 1200 nm thick film similar to that of bulk^7^. The variation of band gap with thickness is in excellent agreement with the theoretical prediction of layer dependency and is in accordance with other reports^4,7,42–45^. The large band gap observed for 50 nm thick film may be due to quantum confinement of layered d-electron dichalcogenides (Se^2−^) and has been observed in other transitional metal dichalcogenides^46–50^.

Room temperature Hall measurements for all the films revealed that SnSe_2_ films are n-type materials with a mobity in the range of µ_e_ = 2.0–8.0 cm^2^ V^−1^ s^−1^, resistivity of ρ = 5–30 Ω cm and carrier concentration of n_e_ = (0.95–6) × 10^17^ cm^−3^. The variation of resisitivity, mobilty and carrier concentration with thin film thickness are shown in Figure S6. It is interesting to note that the resistivity is optimum for a thickness of 700–900 nm, while the mobility peaks around 900 nm. Similarly, the carrier concentartaion is optimum around 700–800 nm. The better connectivity (Figure S7) of grains in the film is believed to be the reason for the better electrical properties. Over all, the mobility and carrier concentration are optimum for 700–900 nm thick films.

**Figure 5:**
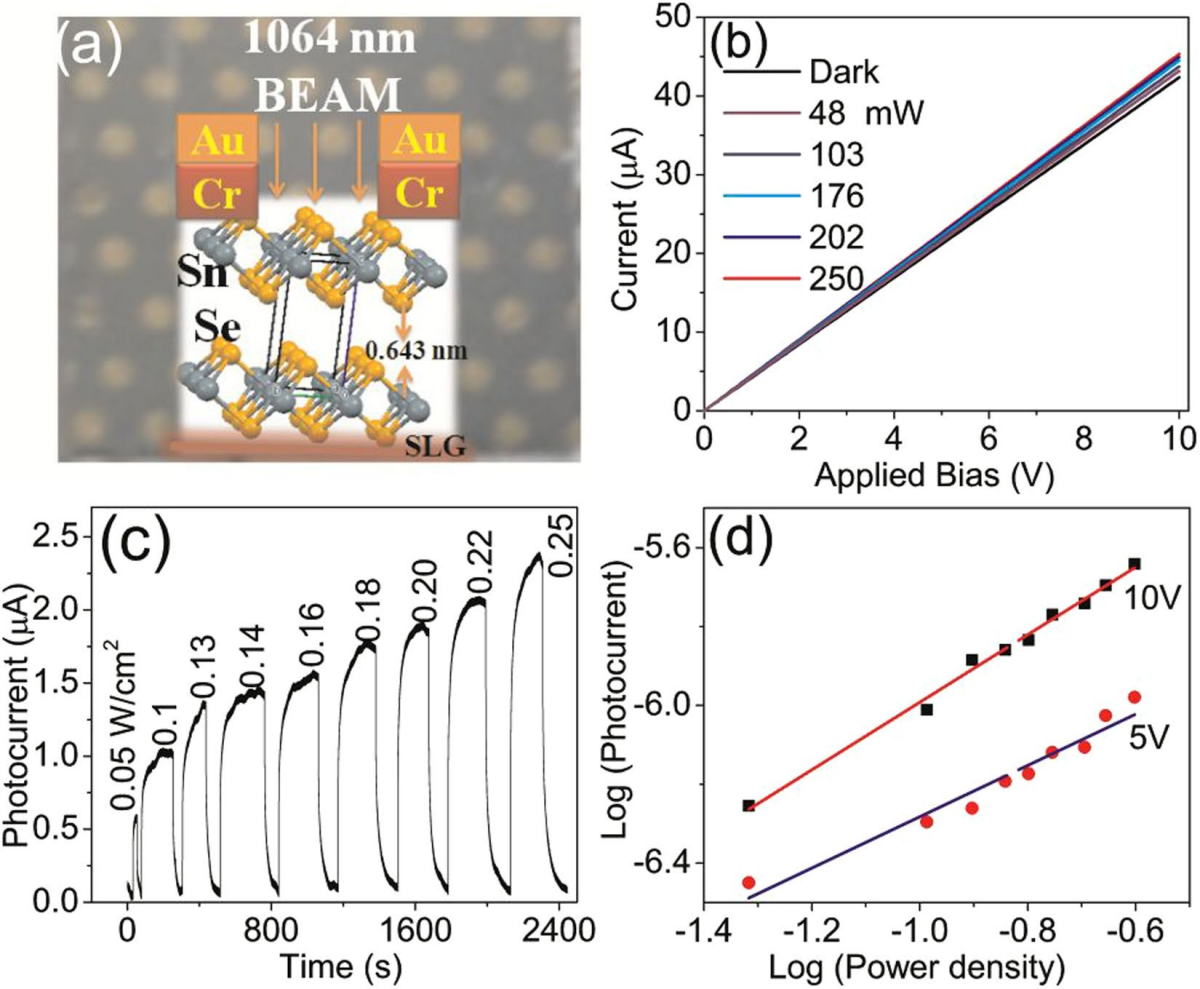
(**a**) Schematic of device architecture with the film in the background, (**b**) *IV* with varying power densities, (**c**) IR (1064 nm) photoresponse of 1200 nm thick SnSe_2_ film with varying power densities under bias voltage of 10 V, and (**d**) Photocurrent versus power density (*I*_Photon_ α *P* ^m^).

It can be noted from Fig. 4d that the band gap of thicker films (>400 nm) is below 1.3 eV, while that of thinner films (<140 nm) is above 1.6 eV. This indicates that thinner films can not be used for IR (1064 nm = 1.165 eV) photodetection. In order to substantiate this speculation, the photoresponse of SnSe_2_ thin films under 1064 nm laser illumination was evaluated using the device configuration shown in Fig. 5a. The optical photograph of the typical device structure is shown in the backgraound. Figure 5b shows the current (*I*)-voltage (*V*) linear behaviour of SnSe_2_ under dark and 1064 nm laser illumination with different power density (0 to 250 mW/cm^2^) and we observe that there is an increase in current upon the laser illumination on 1200 nm thick SnSe_2_ film. The photocurrent is measured under alternating dark and 1064 nm laser light. Figure 5c and S8 show the temporal photoresponse of 1200 nm thick SnSe_2_ film under 1064 nm laser illumination with different power densities upto 250 mW/cm^2^ at a bias voltage of 10 and 5 V, respectively.

It can be noted that the photocurrent increases with increasing bias and power density. The dependence of photocurrent on power density shown in Fig. 5(d) is fitted using the power law^51^: *I*_*ph*_ *α P* ^*m*^ and the exponent *m* which determines the response characteristic of a photodetector with incident power density, is found to increase from 0.7 to 0.9 when the bias is increased from 5 to 10 V. The value of *m* indicates that trap states and interactions between the photogenerated carriers (electron-hole pairs) are involved in the recombination kinetics of photo-carriers^51^ at lower bias and the value of *m* is close to ideal unity value at higher bias which indicates that the photo-generated current can be attributed to efficient separation of electron-hole pairs with less trap states and interaction between photo-generated carriers.

**Figure 6:**
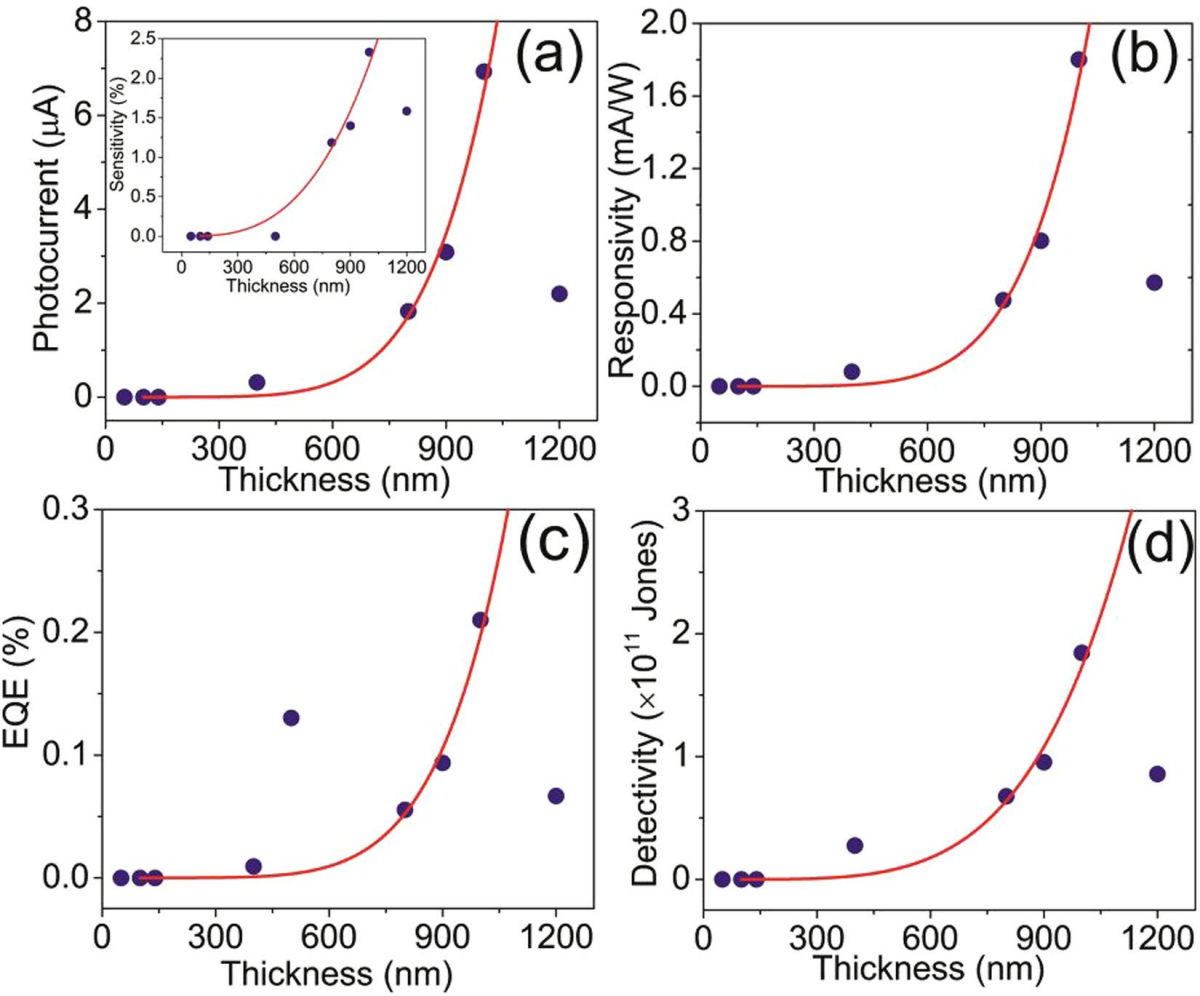
(**a**) Photocurrent with Sensitivity (inset), (**b**) Responsivity, (**c**) EQE, and (**d**) Detectivity versus film thickness at 10 V and 250 mW/cm^2^. The lines are guide to eye.

**Figure 7:**
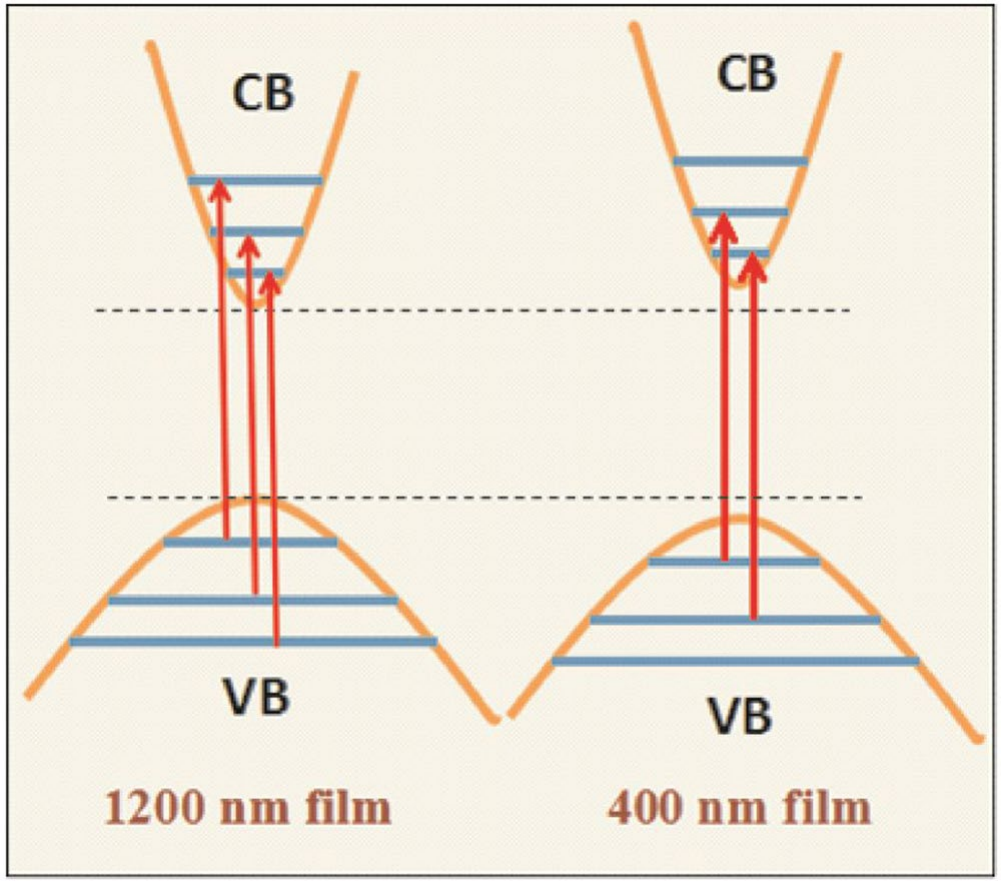
Energy band diagrams for 1200 and 400 nm thick film and the influence of light on the generation of charge carriers.

Photoresponse of different films with a power density of 250 mW/cm^2^ and a bias of 10 V are shown in Figure S9(a–f) and the photocurrent as a function of thickness is shown in Fig. 6(a). The sensitivity (defined as S = I_λ_/I_dark_, I_λ_ = I_light_ − I_dark_, I is the current) as a function of thickness is shown in the inset of Fig. 6(a). It is interesting to note that thinner films do not respond to the 1064 nm irradiation, while the sensitivty increases with thickness for thicker films. As indicated before, the band gap of thinner films is higher than the energy corresponding to 1064 nm and hence, not responsive (Figure S10). In order to testify the band gap dependency, we irradiated 140 nm thick film with white light. Interestingly, the film is found to be sensitive to white light (Figure S11) ensuring the thinner film is insensitive to IR because of the higher band gap. The sensitivity is found to be 90.4% for visible light with 100 mW/cm^2^. Figure 6b shows the variation of responsivity (defined as R_λ_ = I_λ_/(P_λ_A), A is the effective surface area and P_λ_ is the power density. Figure 6c and d show the variation of external quantum efficiency (defined as EQE = hcR_λ_/qλ) and detectivity (defined as D* = R_λ_/(2qI_o_)^1/2^)^52^ with power density. Similar dependency has also been observed for 5 V (Figure S12). It may be noted that R_λ_, EQE and D* increase with thickness as well as with power density (Figure S13). At this point, we would like to note that the photocurrent and sensitivity (Fig. 6(a)) as well as the dark current (Figure S14) increases with the thickness of the films. As the thickness increases the band gap decreases that promotes the enhancement of the darkcurrent. Similarly, the decrease in band gap also promotes more charge carriers to be generated as depicted in Fig. 7 leading to higher photocurrent and hence, the sensitivity.

Figure S15(a–d) shows the ON and OFF IR response characteristic of the device which is well retained even after six cycle repeatations with their respective fitted growth and decay rate constants at 10 and 5 V and are determined by fitting one cycle of the photo-response curve using the second order exponential equation given by I(t)_growth_ = I_dark_ + α exp[t/τ_1_] + βexp[t/τ_2_] and I(t)_decay_ = I_dark_ + χexp[−t/τ_1_] + γ exp[−t/τ_2_ **]** respectively^53^, where α, β, χ and γ are scaling constants, τ_1_ and τ_2_ are time constants, t is the time for ON or OFF cycles and I_dark_ is the dark current. From the fits, we estimate the time constants for growth and decay. When the bias voltage was set to 10 V, the photocurrent rises very fast within 0.38 s upon illumination followed by a slower component of 16 s before saturation. The average response time constant for this process is about 2.5 s. The average time constant was calculated from τ_avearage_ = (ατ_1_ + βτ_2_)/(α + β). After switching off the exciting laser, the photo-current decay follows a second order exponential relaxation process with an estimated time constant of 0.36 and 8.32 s with an average time constant of 3.68 s before reaching the initial dark current. When the bias voltage was 5 V, the response and decay of photoresponse follow a second order exponential relaxation process as is the case of 10 V. The time constants are 0.28 and 15.33 s with an average time constant of ≈7.76 s for the sensor response, while the time constansts for decay are 0.59 and 14.47 s with an average time constant of ≈7.52 s. It can be noted that the fastest response/decay was obtained at a bias voltage of 10 V and is consistent with the power law and is attributed to efficient seperation of electron-hole pairs. The general response is quiet slow as compared to other previously reported SnSe_2_ layered device^15^. This slow response may be due to defects or charge impurity states inside the band gap which act as recombination centres for the photo-generated charge carriers^2,54^. Previous reports with other layered 2D transitional metal dichalogenides have mainly focused on detecting visible light, a narrow range of the electromagnetic spectrum. The cut-off wavelength for our device is 1064 nm which is superior to those obtained by others^15,55–57^ and suggests that SnSe_2_ of about 1200 nm thick grown on SLG can also be used as an efficient IR photodetector. The IR-response and sensitivity of other prepared films in this study are compared in Table S3. Overall, our results suggest that the SnSe_2_ thin film can be explored as an excellent material for photodetection.

## Conclusion

We have fabricated and characterized layered SnSe_2_ thin films of different thickness on SLG substrate by DC sputtering of Sn metal target followed by selenisation. Hall measurements confirm that SnSe_2_ is n-type material with carrier mobility between 2.0–8.0 cm^2^ V^−1^ s^−1^, resistivity between 5–30 Ω cm and carrier concentration n_e_ between (0.95–6) × 10^17^ cm^−3^. We have observed that the band gap depends on thin film thickness. For 50 nm thick film, the band gap is ~2.04 eV similar to that of monolayer, whereas it is approximately 1.2 eV for the 1200 nm thick film similar to that of bulk. The IR photodetection response of SnSe_2_ demonstrates a sensitivity of ~3% for a 1000 nm thick film and the response time constant is 0.38 s at a bias of 10 V. On the other hand, the sensitivity is 90.4% for 140 nm thick film at 100 mW/cm^2^ which is insesitive to IR. Overall, our results suggest that the SnSe_2_ thin film can be explored as an excellent material for photodetection and the sensitivity with response time can be improved further by increasing the applied bias.

## Methods

### Thin film Deposition

**Figure 8:**
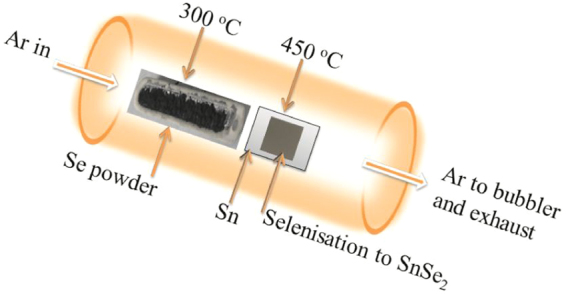
Schematic of a temperature profile inside the tubular furnace with an inner diameter of ~5.2 cm and outer diameter of ~6.2 cm.

Prior to deposition, soda lime glass substrates (SLG) were cleaned by placing them in dilute HCl for 10 minutes, followed by sonication in de-ionised (DI) water and then boiled in iso-propanol alcohol (IPA) at 82.5 °C for about 15 minutes. The substrates were then purged with nitrogen gas to remove any contaminants such as water vapour. The vaccum chamber was first evacuated to a base pressure of about 5.0 × 10^−6^ mbar. Sn was then sputtered onto SLG between 15–47 W for 5–20 minutes using argon as plasma source flowing at 1.5 sccm. The chamber working pressure was maintained at 4.0 × 10^−3^ mbar throughout the deposition to obtain films of various thicknesses ranging from 30 to 600 nm. The substrate was maintained at 20 rotations per minute to obtain films of uniform thickness. The Sn films on SLG was annealed in a calibrated tube furnace as shown in Fig. 8. 1 g of Se powder in Alumina boat was placed in a zone with temperature of about 300 °C (above the melting point of Se), whereas Sn thin films on SLG was placed in a temperature zone of about 450 °C. Prior to selenization process, argon gas was passed through the tube furnace for 5 minutes to drive out the oxygen gas present. The tube furnace ramp rate was maintained at 3 °C per minute to a temperature of 450 °C and held constant for 1 h. The furnace was cooled down to room temperature naturally. Argon gas flow was maintained at 20 sccm throughout the annealing and cooling processes.

### Thin film Characterization

The crystal structure of the films have been accessed by X-ray diffraction (XRD) using CuK_α_ (1.5418 Å) (X’pert-PRO PANAlytical instruments). The crystalline properties of the film were further determined using 200 kV FETEM (JEM-2100F) and were prepared by scratching the film surface. The powder collected was dispersed in IPA, sonicated for about 10 minutes and dropped onto carbon coated copper grids. The surface morphology of the films, roughness and thickeness were determined using non-contact mode AFM (A.P.E. Research A100-AFM). Veeco Dektak 6 M surface profilometer was used to measure the thickness of the film. Diffuse Reflection Spectrum (DRS) of the thin film was obtained using UV-Vis-NIR spectrophotometer (Perkin Elmer-lambda 750 Instruments). Raman study was carried out at room temperature in the range of 50–600 cm^−1^ using Visible LabRAM HR instruments with a 532 nm laser. X-ray photoelectron spectroscopy (XPS) measurements were performed using AXIS Ultra DLD X-ray photoelectron spectrometer with MgK_α_ X-ray source. The system was maintained in ultra-high vacuum at a base pressure of 6.8 × 10^−9^ Torr. The C1s peak at 284.60 eV was taken as reference to correct the binding energy values of our samples.

### Device fabrication and current-voltage measurements

The electrical contacts of Cr/Au (6 nm/80 nm) was thermally evaporated and deposited on top of SnSe_2_ thin films. The current-voltage characteristics were measured using a Keithley SMU2400 source meter and 1064 nm laser with varrying intensity. The Hall measurements were conducted at room temperature in the presence of 0.55 T magnetic field using Ecopia HMS 5000 Hall effect measurement system and measurements were taken in the Van der Pauw geometry.

### Data Availability

The datasets generated during and/or analysed during the current study are available from the corresponding author on reasonable request.

## Electronic supplementary material

Supplementary Information
