# Supplementary material for: Band Gap Engineering of Hexagonal SnSe2 Nanostructured Thin Films for Infra-Red Photodetection
Source: Sci Rep. 2017 Nov 9;7:15215. doi: 10.1038/s41598-017-15519-x (PMC5680184; doi:10.1038/s41598-017-15519-x)
Supplement: Supplementary file 1 — Supplementary Information [file 41598_2017_15519_MOESM1_ESM.pdf]

Band Gap Engineering of Hexagonal $SnSe_2$ Nanostructured Thin Films for Infra-Red Photodetection

*Emma P. Mukhokosi, Saluru B. Krupanidhi and Karuna K. Nanda**

*E-mail: nanda@mrc.iisc.ernet.in

Materials Research Center, Indian Institute of Science, Bangalore-560012, India

Supplementary Information (SI)

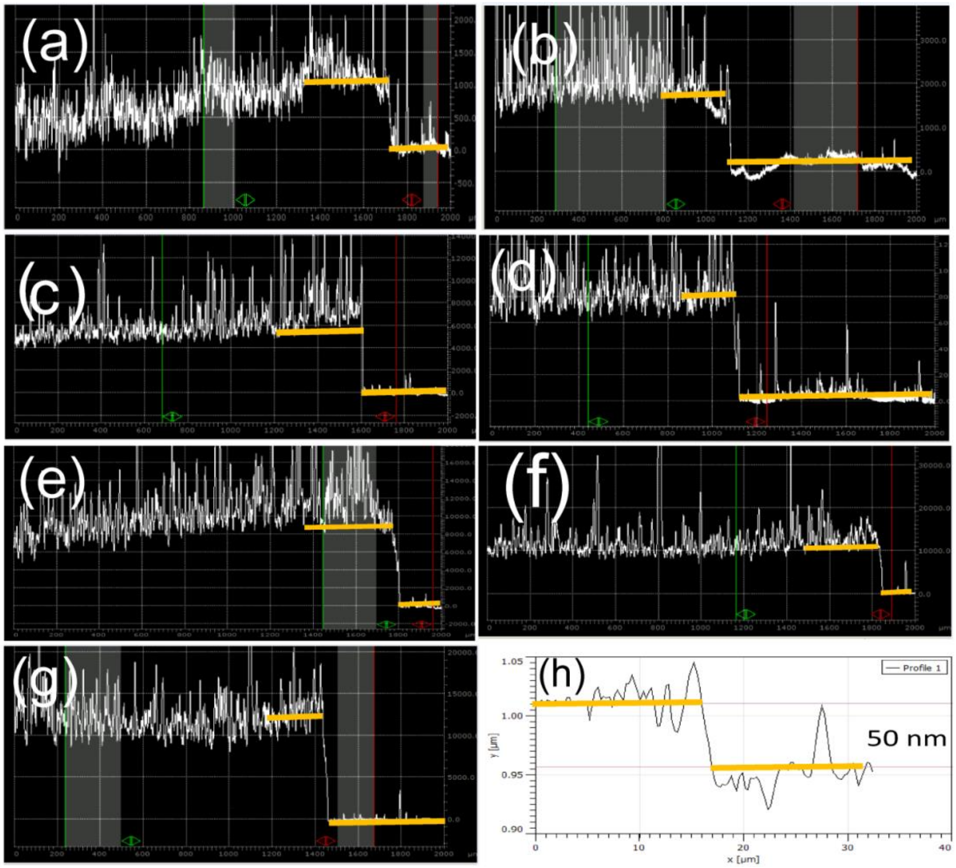

Figure S1. (a-g) $SnSe_2$ film thickness of 100,140, 500, 800, 900, 1000 and 1200 ± 10 nm respectively taken from Dektak measurements, and (h) film thickness of 50 nm taken from AFM.

Rietveld refinement parameters

The least squares refinement is based on the following equations; $R_p = \frac{\sum|Y_{o,i} - Y_{c,i}|}{Y_{o,i}}$,

$$R_{wp} = \sqrt{(\frac{\sum w_i (Y_{o,i} - Y_{c,i})^2}{\sum w_i Y_{o,i}^2})}, R_B = \frac{\sum| I_{(o,k)} - I_{(c,k)} |}{\sum I_{(o,k)}}, R_F = \frac{\sum| I_{(o,k)}^{1/2} - I_{(o,k)}^{1/2} |}{\sum I_{(o,k)}^{1/2}}, \chi^2 = (\frac{R_{wp}}{R_{exp}})^2$$

where R_p , R_{wp}, R_B, R_{exp}, and χ^2 are the R-pattern, R-weighted pattern, R-Bragg factor, R-expected and goodness of fit respectively. $Y_{o,i}$ and $Y_{c,i}$ are the observed and calculated data, respectively at data point i, N is the number of data points, P is the number of parameters, w_i is the weighting given to data point i. $I_{o,k}$ and $I_{c,k}$ are the observed and calculated intensities of the k th reflection. The obtained lattice parameters and indicators of the quality of refinement are shown in **Table S1 (XRD refinement parameters)**. The strain (ε) was obtained from Williamson-Hall plot based on Bcos(θ) = kλ/L + 4×sin(θ), where B(FWHM) is the the crystallite line broadening, k (Scherrer constant) varies from 0.6 - 2.0[1] and was identified as 0.74 and λ = 1.5418 Å. From Rietveld analysis, the position of atoms in the unit cell and their Wyckoff positions are evaluated and provided in **Table S2**. It contains 1Sn and 2Se atoms in 1a and 2d Wyckoff positions (Wp), respectively.

Table S1. XRD refinement parameters.

χ2 (GOF)	R_{wp}(%)	R_p(%)	a (Å)	b (Å)	c (Å)	γ (°)	XRD size (nm)	ε(%)
7.12	5.12	3.27	3.818	3.818	6.152	120	186	0.12

Table S2. Fractional coordinates of atoms in the unit cell, occupancy and their Wyckoff positions (Wp).

Element	Fractional coordinates	Occupancy	Wp
Sn^{4+}	(0.000, 0.000, 0.000)	1.5	1a
Se^{2-}	(0.3333, 0.6667, 0.2217)	1.0	2d

Raman spectra:

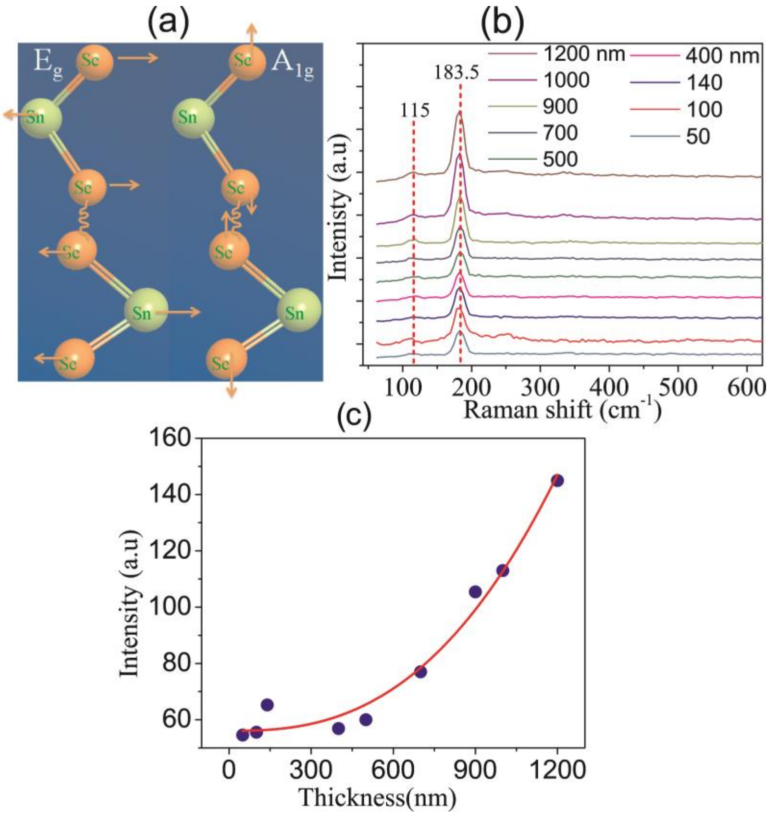

Figure S2. (a) Schematic diagram indicating the out of plane vibration mode (E_g) and in-plane (A_{1g}) vibration mode of $SnSe_2$. (b) Raman spectra of $SnSe_2$ films. (c) The variation of intensity of A_{1g} mode with thickness.

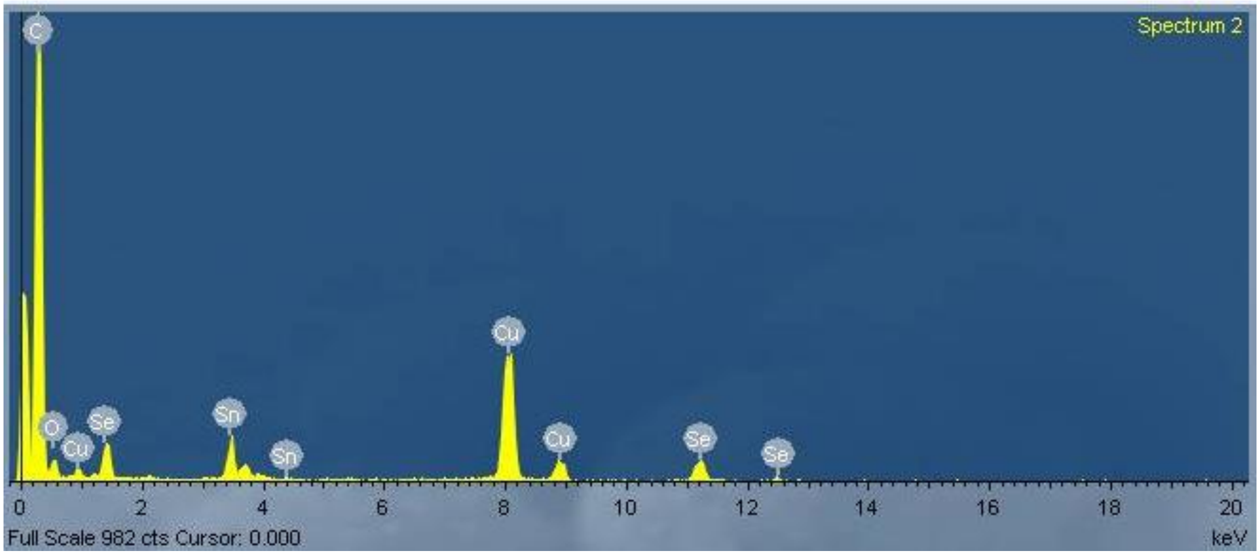

Figure S3. EDS of 1200 nm thick $SnSe_2$ film.

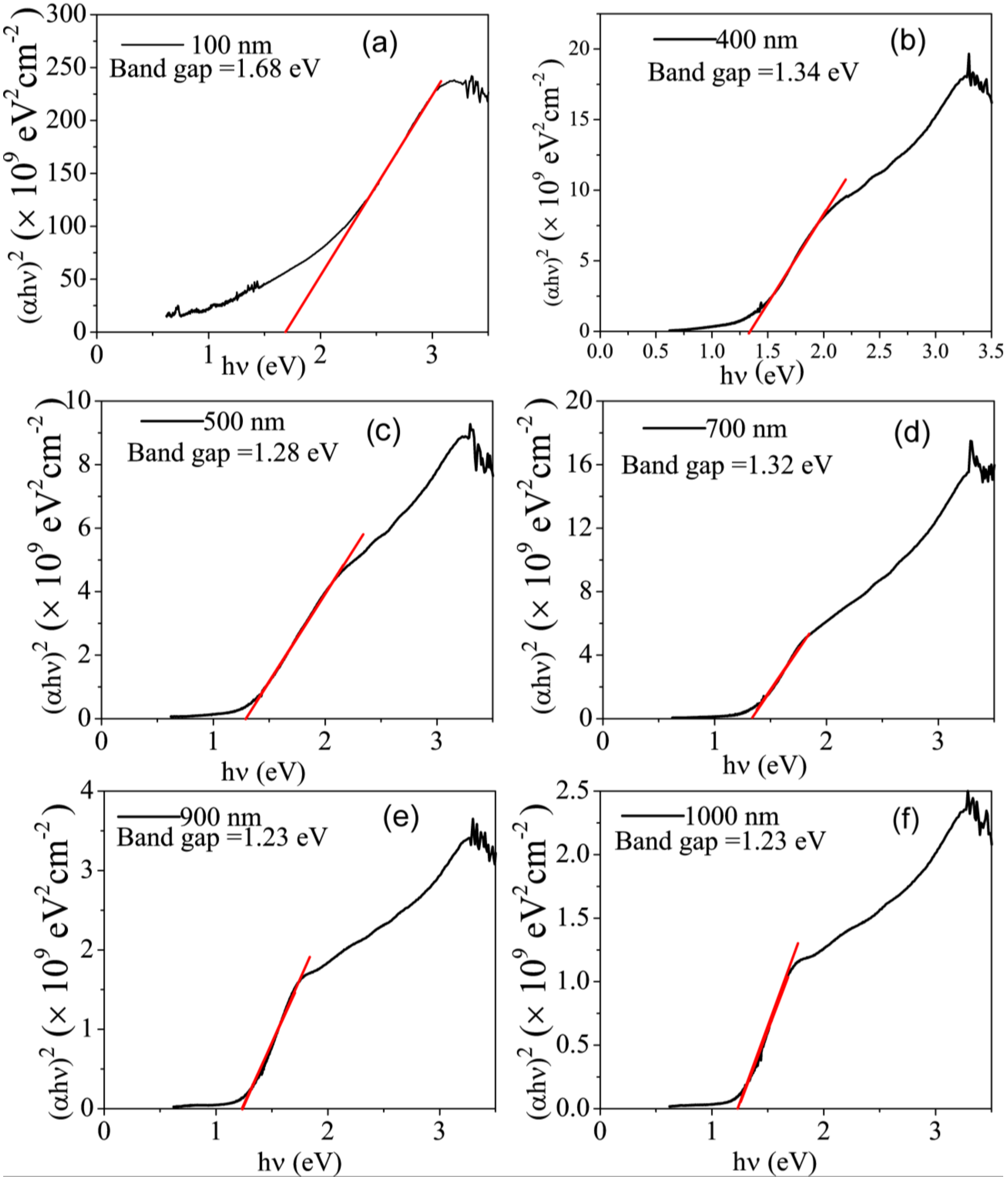

Figure S4. (a-i) Tauc plots for 100, 400, 500, 700, 900 and 1000 nm thin films, respectively.

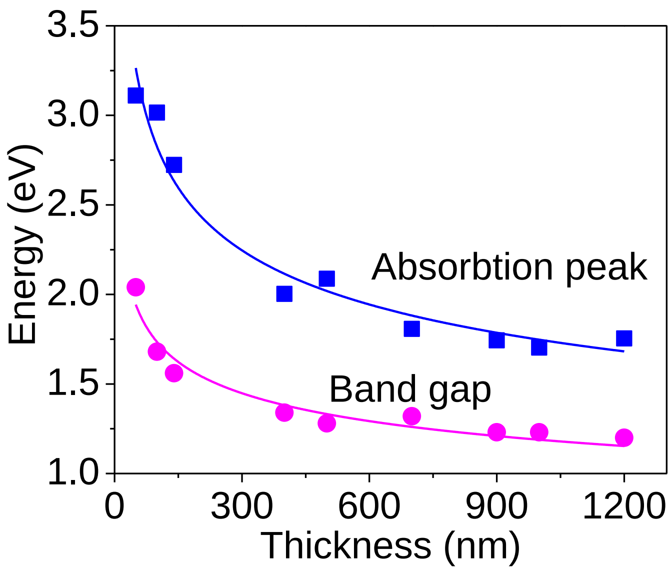

Figure S5. Variation of band gap and absorption peak energy with film thickness.

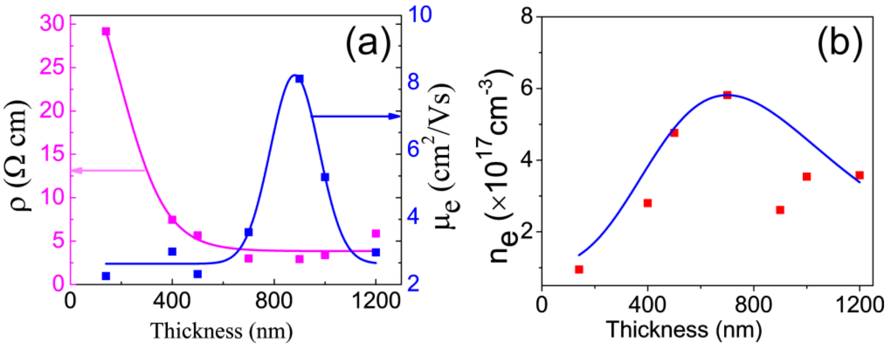

Figure S6. (a) Room temperature variation of resistivity and mobility, and (b) carrier concentration with film thickness.

AFM images $SnSe_2$ thin films: Two-dimensional (2-D) AFM images of all the films are shown in **Figure S7** and reveal that the polycrystalline nature of the films. The surfaces of annealed $SnSe_2$ thin films are rough with rms (root mean square) roughness between 50-136 nm.

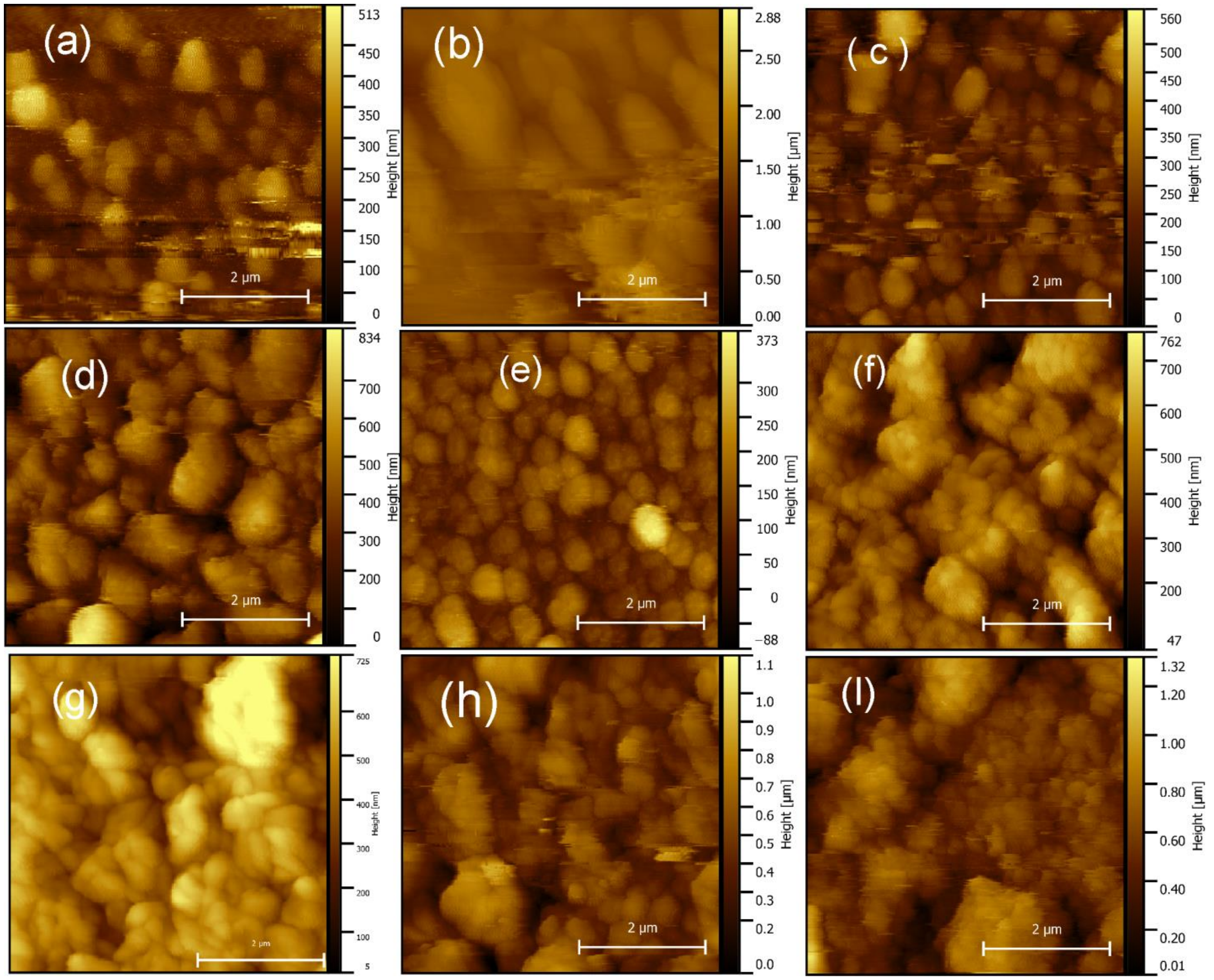

Figure S7. (a-i) 2-D AFM images of 50-1200 nm thick $SnSe_2$ thin films.

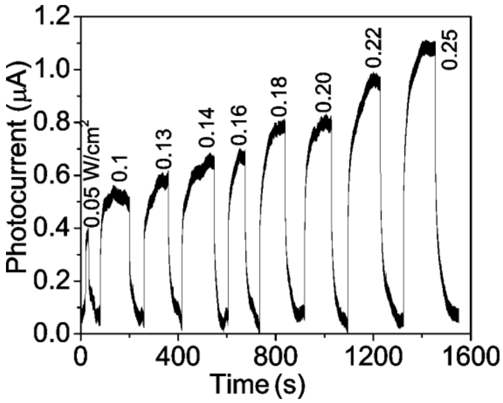

Figure S8. Power dependent photo-current of 1200 nm thick film under bias voltage of 5 V.

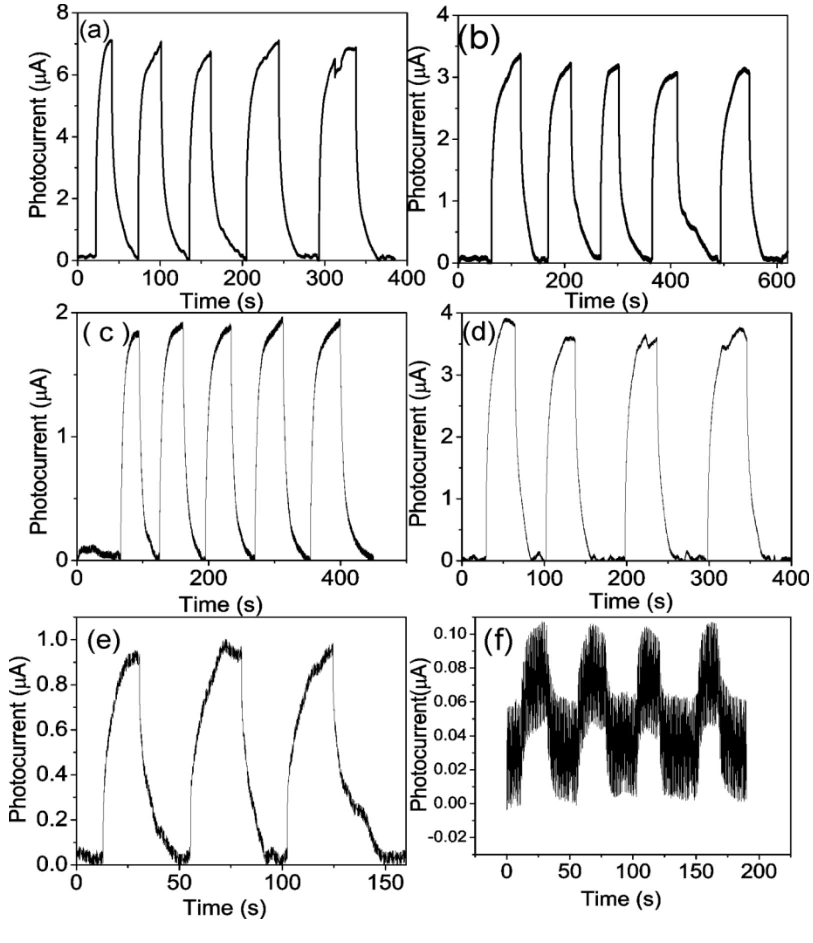

Figure S9. (a-f) Photoresponse of films with 1000, 900, 800, 500, 400 and 140 nm thick films at power density of 250 mW/cm^2 and a bias voltage of 10 V.

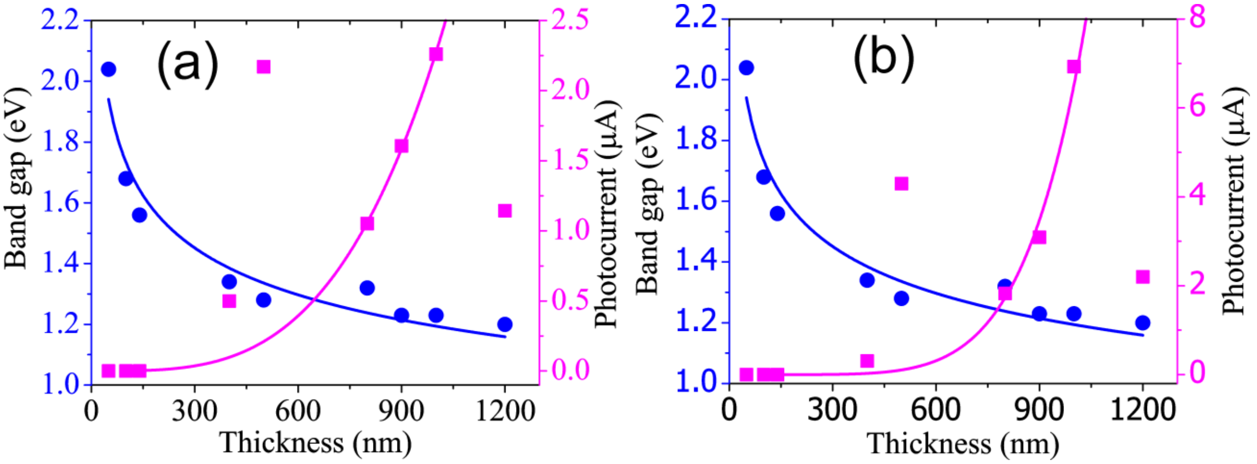

Figure S10. Band gap and photocurrent at a bias of (a) 5 V and (b) 10 V) as a function of film thickness. The power density is 250 mW/cm^2.

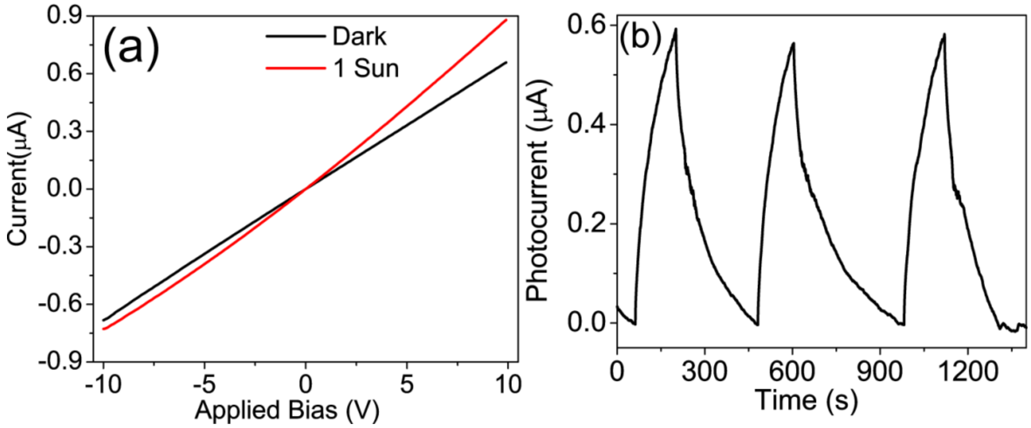

Figure S11. (a) *I-V* and (b) Photoresponse of 140 nm film under solar simulator with 1 sun (100 mW/cm^2). The sensitivity is 90.4% for visible light, while it is close to zero for 1064 nm with 250 mW/cm^2.

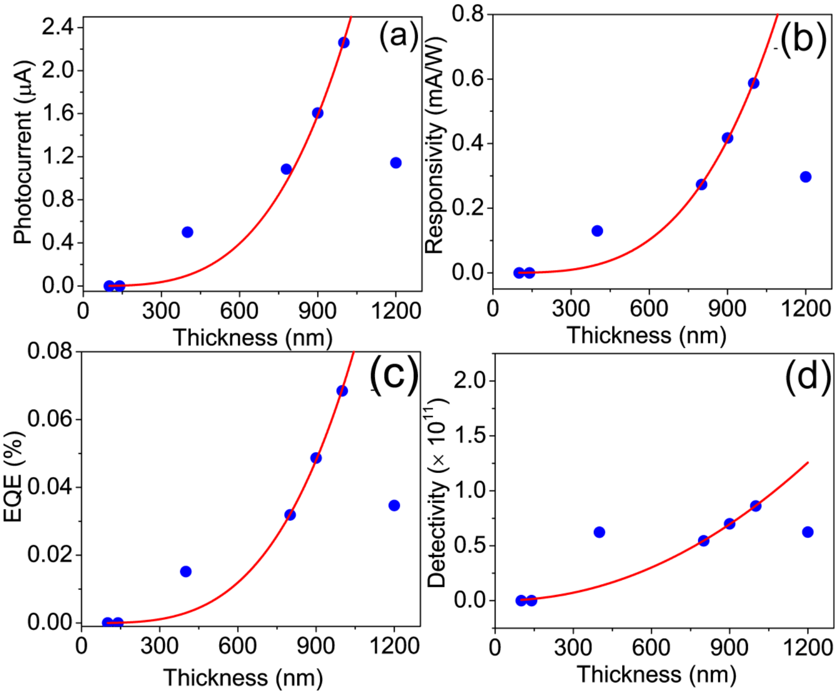

Figure S12. (a) Photocurrent, (b) Responsivity, (c) EQE, and (d) Detectivity versus film thickness at 5 V and 250 mW/cm^2. The lines are guide to eye.

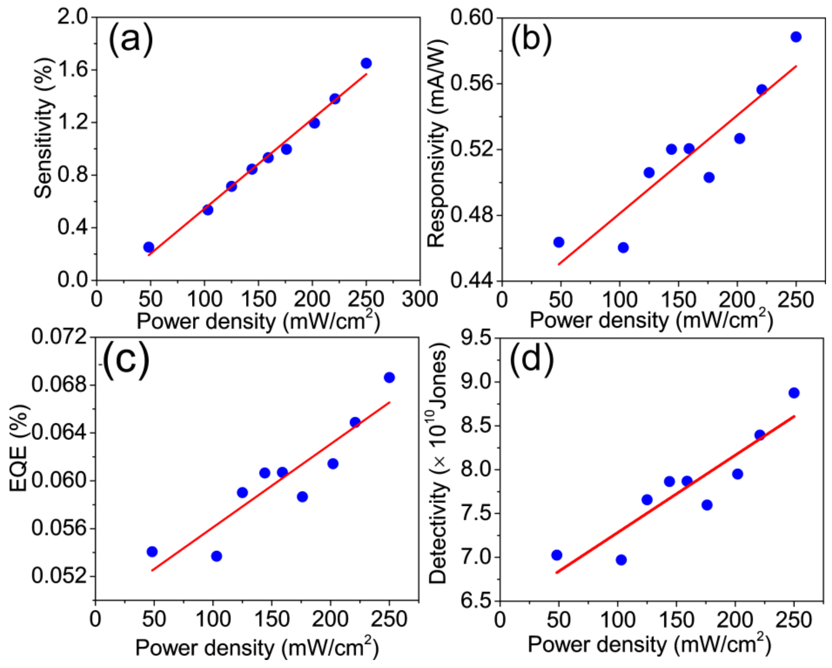

Figure S13. (a) Sensitivity, (b) Responsivity, (c) EQE, and (d) Detectivity versus power density at 5 V bias for 1200 nm thick film. The lines are guide to eye.

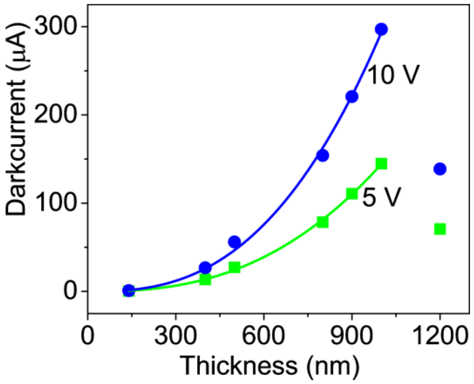

Figure S14. Dark current vs thickness at 5 and 10 V .

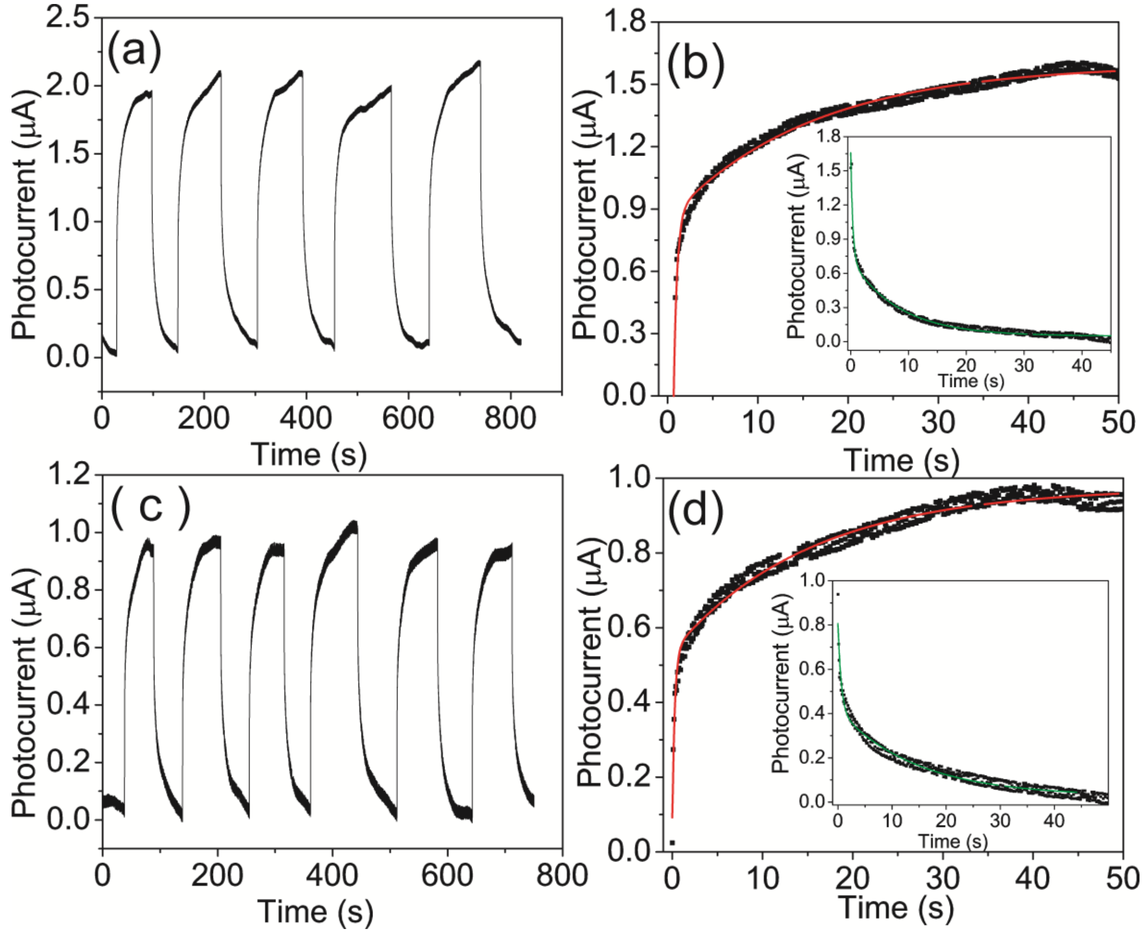

Figure S15. Photoresponse under bias voltage of (a and b) 10 and (c and d) 5 V with power density of 250 mW/cm^2 and their corresponding fitted growth and decay (insets of b and d) curve for 1200 nm thick $SnSe_2$ film.

Table S3. Comparison of photoresponse parameters.

Material	Method	Bias (V)	$\lambda_{cutt-off}$ (nm)	R_λ (mA/W)	EQE (%)	D*(Jones)	Response time	Ref
Quartz/ WS_2/Ti/Au	CVD	5.0	514	$9.2x10^{-2}$	-	-	5.3 ms	2
Si/Al_2O_3/ MoS_2/Au/Ti	Mechanical exfoliation		633	150	-	-	-	3
Si/SiO_2/$MoSe_2$/Au	CVD	10	532	13	-	-	60 ms	4
SLG/Mo/$CuIn_{(1-x)}$ Al_xSe_2/Al	DC Sputtering	4	790	490	-	-	-	5
Al/Cu(In,Al)Se_2/Al	DC Sputtering	3	790	520	-	-	-	6
Si/SiO_2/$SnSe_2$/Ti/Au	CVD	3	530	$1.1x10^6$	2.6×10^5	10^{10}	14.5 ms	7
Si/CdTe/Au	RF Sputtering	-	A.M. 1.5G	80-500	-	-	< 1s	8
SLG/$SnSe_2$/Cr/Au	DC Sputtering	5	1064	0.6	0.07	1.0×10^{11}	0.28 s	**Present**
SLG/$SnSe_2$/Cr/Au	DC Sputtering	10	1064	2	0.2	2.0×10^{11}	0.38 s	**Present**

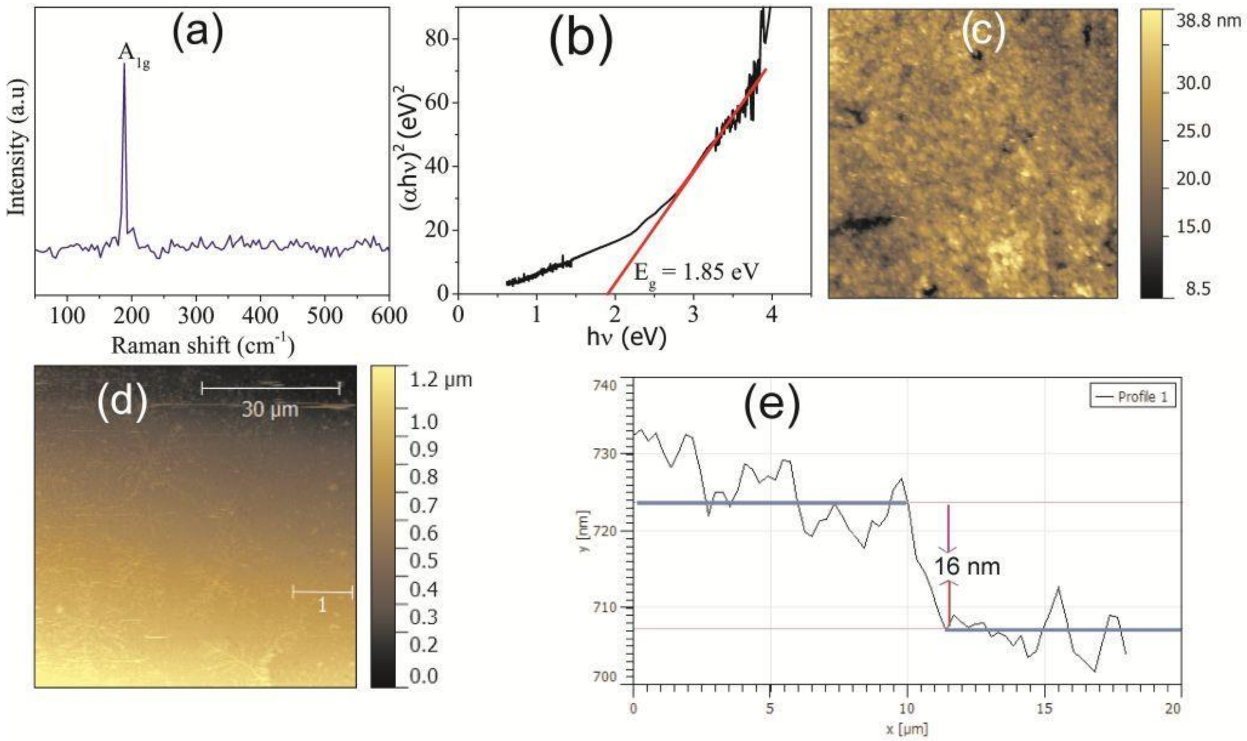

Figure S16. (a) Raman spectrum, (b) Tauc plot, (c) 2D AFM image, (d) 2D AFM image with profile 1 between sample and substrate where step height was taken and (e) step height indicating ~16 nm thin film.

The thinnest sample that has been realized by this two-step process is shown in **Figure S16** obtained by DC sputtering of Sn target at 10W for one minute. Only the A_{1g} Raman active mode located at 188.5 cm^{-1} could be identified as shown in **Figure S16(a)**. Other samples with thickness between 50 to 1200 nm have A_{1g} mode located at 183.5 cm^{-1}. Similar kind of shift has been observed in other transitional metal dichalogenides such as MoS_2[9,10] and it is attributed to Columbic interaction and possible stacking-induced change of the intra-layer bonding.[11] The optical band gap of the film was estimated from Tauc plot as ~ 1.85 eV (**Figure S16(b)**), similar to that of 50 nm film. The morphology and thickness of the sample is presented in **Figure 16 (c-d)** which clearly reveals a thickness of 16 nm. In this context, it may be noted that 16 nm of thick

film is realized by depositing 7 nm thick Sn film by sputtering with a power of 10 W for 1 minute.

References

1. Langford, J. I. & Wilson, A. J. C. Scherrer after sixty years: A survey and some new results in the determination of crystallite size. *J. Appl. Crystallogr.* **11,** 102–113 (1978).

2. Perea-Lõpez, N. *et al.* Photosensor device based on few-layered WS_2 films. *Adv. Funct. Mater.* **23,** 5511–5517 (2013).

3. Choi, W. *et al.* High-detectivity multilayer MoS_2 phototransistors with spectral response from ultraviolet to infrared. *Adv. Mater.* **24,** 5832–5836 (2012).

4. Xia, J. *et al.* CVD synthesis of large-area, highly crystalline $MoSe_2$ atomic layers on diverse substrates and application to photodetectors. *Nanoscale* **6,** 8949–8955 (2014).

5. Chang, R.-P. & Perng, D.-C. Near-infrared photodetector with $CuIn_{1-x}Al_xSe_2$ thin film. *Appl. Phys. Lett.* **99,** 81103 (2011).

6. Chang, R. P. & Perng, D. C. Nano-structured $Cu(In,Al)Se_2$ near-infrared photodetectors. *Thin Solid Films* **529,** 238–241 (2013).

7. Zhou, X. *et al.* Ultrathin $SnSe_2$ Flakes Grown by Chemical Vapor Deposition for High-Performance Photodetectors. *Adv. Mater.* **27,** 8035–8041 (2015).

8. Akgul, F. A., Akgul, G., Gullu, H. H., Unalan, H. E. & Turan, R. Enhanced diode performance in cadmium telluride-silicon nanowire heterostructures. *J. Alloys Compd.*

644, 131–139 (2015).

9. Li, H. *et al.* From bulk to monolayer MoS_2: Evolution of Raman scattering. *Adv. Funct. Mater.* **22,** 1385–1390 (2012).

10. Zhao, Y. *et al.* Interlayer breathing and shear modes in few-trilayer MoS_2 and WSe_2. *Nano Lett.* **13,** 1007–1015 (2013).

11. Huang, X., Zeng, Z. & Zhang, H. Metal dichalcogenide nanosheets: preparation, properties and applications. *Chem. Soc. Rev.* **42,** 1934 (2013).
